# Additive effect of patient anosognosia and theory of mind deficit on dementia caregiver distress

**DOI:** 10.3389/fneur.2026.1724172

**Published:** 2026-03-26

**Authors:** Manizhe Eslami-Amirabadi, Aaron Scheffler, Joel H. Kramer, Maria Luisa Gorno-Tempini, William W. Seeley, Howard Rosen, Gil D. Rabinovici, Bruce L. Miller, Winston Chiong, Katherine P. Rankin

**Affiliations:** 1 Department of Neurology, Memory and Brain Wellness Center, University of Washington School of Medicine, Seattle, WA, United States; 2 Department of Neurology, Edward and Pearl Fein Memory and Aging Center, University of California, San Francisco, San Francisco, CA, United States

**Keywords:** anosognosia, caregiver distress, dementia, neuroethical considerations, theory of mind deficit

## Abstract

**Introduction:**

Caregiver distress in dementia is multifactorial. The contribution of disease specific factors including anosognosia (poor awareness of cognitive/behavioral deficits) and theory of mind (ToM) deficit (difficulty with understanding other’s perspective) requires further investigation.

**Method:**

Cross sectional secondary analysis was performed on a dataset of 205 research participants (age = 64.2 ± 9.46): 57 Alzheimer’s disease, 38 behavioral variant frontotemporal dementia, 12 non-fluent primary progressive aphasia (PPA), 24 semantic variant PPA, 18 progressive supranuclear palsy syndrome, 14 corticobasal syndrome, and 42 cognitively normal controls (NC). Anosognosia was measured using the Patient Competency Rating Scale (PCRS-self minus PCRS-caregiver; clinically meaningful anosognosia >20 points difference), ToM deficit was evaluated using The Awareness of Social Inference Test: Social Inference-Enriched (TASIT-SIE), and caregiver distress was measured using the Neuropsychiatric Inventory Questionnaire (NPI-Q) Total Distress score. Differences across syndromes were evaluated controlling for age and sex, and multivariable linear regression was used to determine predictors of caregiver distress.

**Results:**

Clinically meaningful anosognosia (patient overestimation of function) and ToM deficit were significantly higher in all dementia syndromes compared to NCs. Anosognosia and ToM deficit each independently predicted caregiver distress and had an additive effect (*p* < 0.05).

**Discussion:**

Our findings are consistent with other research showing that anosognosia in individuals with chronic neurological disorders including dementia can increase caregiver distress; however, our results highlight the additive importance of patients’ theory of mind deficits above and beyond their anosognosia. Evaluation of both patient anosognosia and ToM deficit in clinical contexts may provide meaningful information to predict which caregivers are at particular risk for adverse outcomes.

## Introduction

Dementia is a growing public health problem associated with many adversities for affected individuals, their care partners, and society. Currently, more than 7 million American above 65 years old are living with dementia in the US and with the population aging, the toll of dementia is likely to increase. They currently require nearly 12 million unpaid caregivers whose labor is equal to $413 billion of estimated value (1). Moreover, the cost of early onset dementia (onset< 65 years old) is estimated to be double the cost of late onset dementia (2). Dementia is associated with loss of independence and shorter lifespan for patients (1) and causes significant distress for caregivers (3). Taken together, these highlight the significance of the efforts needed to comprehend every aspect of this serious illness to address it appropriately and consequently improve outcomes.

While many neurodegenerative diseases primarily affect systems mediating memory, language, or movement, evidence is mounting that even early in the disease process, behavior and social cognition changes occur in most if not all neurodegenerative syndromes. More specifically, anosognosia (poor awareness of cognitive/behavioral deficits) and Theory of mind (ToM) deficit (difficulty with understanding others’ perspective) are frequently observed in almost all dementia syndromes as follows. Alzheimer’s disease (the most common dementia syndrome characterized by memory loss with variable combination of other cognitive symptoms including executive dysfunction, visuospatial difficulties, language deficits due to amyloid and tau accumulation) (4) was shown to be associated with anosognosia (5–7), even in early stage of mild cognitive impairment (8) and ToM deficit (9–11). Frontotemporal Dementia (FTD) syndromes that include a heterogenous group of disorders (mostly due abnormal tau and TDP-43 accumulation) with various clinical presentations were also shown to be associated with anosognosia (12–14) and ToM deficit (9, 10, 15–17). The main FTD clinical syndromes are as follows:

- Behavioral variant FTD (bvFTD) where the hallmark symptoms are apathy, lack of empathy, disinhibition and ritualistic behavior (18).
- Corticobasal syndrome (CBS) with prominent and often asymmetric parkinsonism associated with early cognitive deficit (19).
- Progressive Supranuclear Palsy syndrome characterized by symmetric akinetic parkinsonism with varying degree of supranuclear eye movement deficit and cognitive impairment (20).
- Nonfluent primary progressive aphasia (nfvPPA) that primarily affects motor speech and grammar (21).
- Semantic variant PPA (svPPA), which degrades semantic knowledge and results in significant impairments in understanding and producing language and appropriate social behavior (21).

Caregiver distress is an important factor in predicting the outcomes for the future wellbeing of people living with dementia (PLwD) and caregivers themselves. A systematic review including 81 studies on the topic (*n* = 43,761) showed that caregiver distress can cause worsening behavioral and psychological symptoms of dementia, increased rates of elder abuse, and institutionalization of the care recipient (22). Additionally, a scoping review identified studies showing that caregivers of PLwD experience worse psychological (depression, anxiety symptoms) and physical outcomes (increased risk of cardiovascular disease, sleep disturbance, respiratory symptoms, joint pain) and more substance use disorder (especially alcohol in the US) compared to non-caregivers and non-dementia caregivers (23). On the other hand, studies have shown improvement of caregiver distress with various caregiver-focused psychosocial interventions (24, 25) [guided DICE method (26) recommendations for dementia symptom management, providing dementia specific education, web-based daily messages to encourage communication and to provide support as needed] suggesting it is an important modifiable factor in health and wellbeing of PLwD and their caregivers. Therefore, it is critical to better understand the factors contributing to caregiver distress in dementia care.

The distress attributed to caregiving in dementia is multifactorial including patient-specific symptoms (such as anosognosia and ToM deficit) and caregiver-specific factors [including psychosocial factors (coping style, mental health) (27), household income (28), and cohabitation with the PLwD (29) play a role in the caregivers’ experience]. Anosognosia and ToM are symptoms of dementia that cause difficulty with communication between the patient and their caregiver. They are separately shown to be associated with increased caregiver distress. The contribution of anosognosia in increasing caregiver distress has predominantly been studied in mild cognitive impairment (MCI) (30), Alzheimer’s disease syndrome (AD) (31), and behavioral variant of frontotemporal dementia (bvFTD) (24). Theory of mind (ToM) deficit is also shown to worsen caregiver distress mostly in AD and bvFTD (10). Additionally, anosognosia (32) and ToM deficit (33) were shown to worsen neuropsychiatric symptoms of dementia (anxiety, agitation, disinhibition, irritability, apathy) that are themselves associated with more caregiver burden (3, 24). Despite their important effect on caregiver distress, whether anosognosia and ToM deficit interact to have an additive negative impact on caregiver distress is unknown. Furthermore, the role of anosognosia and ToM deficit in other dementia syndromes such as the primary progressive aphasias, corticobasal syndrome and progressive supranuclear palsy syndrome is not well investigated. Thus, for this study we measured caregiver distress alongside of patient anosognosia and ToM deficit in a large sample of individuals with those neurodegenerative syndromes to investigate the differential impact and interplay of these patient factors on caregiver distress in the context of different dementia presentations.

## Materials and methods

### Participants

In this cross-sectional study, we included 163 participants and their caregivers, including patients with the following neurocognitive disorders, diagnosed according to appropriate international consensus criteria: 57 Alzheimer’s disease syndrome (AD) (4), 38 behavioral variant of frontotemporal dementia (bvFTD) (18), 14 corticobasal syndrome (CBS) (19), 18 progressive supranuclear palsy syndrome (20), 12 with clinical diagnosis of non-fluent variant primary progressive aphasia (21), “MCI-language” (mild cognitive impairment due to unspecified language deficit) or “unspecified primary progressive aphasia” (we categorized them as “PPA” in this paper) and 24 semantic variant primary progressive aphasia (svPPA) (21). We categorized individuals with the logopenic variant of primary progressive aphasia syndrome within the AD group because this diagnosis has a high predictive value for AD pathology when made by specialized speech pathologists and behavioral neurologists in tertiary centers like UCSF-MAC (34). We also included 42 cognitively normal controls (NC) older adults enrolled in the studies of aging for comparison (total of 205 participants; complete sample of participants who had measures needed for our study). All patients underwent a thorough diagnostic evaluation, including complete clinical history, neurologic examination, neuroimaging, neuropsychological evaluation, and functional/neuropsychiatric evaluation to clarify their dementia syndrome. The final diagnosis of dementia syndrome was made in a multidisciplinary clinical consensus conference. While a large proportion of the data were collected before amyloid biomarkers were available, a subset of participants with AD syndrome had confirming evidence of amyloid pathology via amyloid PET imaging or CSF biomarkers. All NCs were evaluated with a similar comprehensive evaluation including neurologic evaluation, neuroimaging, and neuropsychological testing and were included only if they showed typical cognitive performance, did not have any neurologic, primary psychiatric, or significant medical illnesses, and had Clinical Dementia Rating (CDR) score of zero. Individuals were included if they met criteria for the syndromes of interest, had completed a measure of anosognosia [the Patient Competency Rating Scale, PCRS (35)]. Because dementia stage (measured by CDR) was highly likely to mediate the association between dementia and anosognosia (36), we only included participants at the very mild stage of their neurodegenerative condition (CDR < =1) to homogenize our sample on this factor.

### Ethical considerations

After full evaluation of their capacity to provide consent, a written informed consent was obtained from all participants in consultation with their caregivers (for patients). Human procedures followed Helsinki guidelines and were approved by the University of California San Francisco Committee on Human Research. The consent numbers for this study were UCSF #460232 and #458765.

### Measurements

#### Caregiver distress

We used the caregiver distress portion of the Neuropsychiatric Inventory Questionnaire (NPI-Q) as a measure of dementia caregiver distress. NPI-Q collected from study partners close to the time of Patient Competency Rating Scale (PCRS) assessment. NPI-Q is a validated informant-based interview that assesses neuropsychiatric symptoms (including resistance to care: “Is the patient resistive to help from others at times, or hard to handle?”) in various neurological disorders, such as dementia (37, 38). After asking about the presence and severity of each neuropsychological symptom, the informant is asked to rate their distress level related to that symptom on a 5-point scale (0 = not distressing at all, 5 = extremely distressing, unable to cope with). We used the total distress score calculated by adding the distress scores from the 12 questions and created a distress score ranging from 0 to 60.

#### Anosognosia

We used the Patient Competency Rating Scale (PCRS) (35, 39) Questionnaire to measure anosognosia, defined as the discrepancy between participants and their study partners in rating the participants’ cognitive and functional abilities. The questions explore basic and instrumental activities of daily living, emotional control, cognitive and interpersonal abilities. The PCRS questionnaire has 2 components:

- PCRS-self: elicits the participants’ perspective on their own cognitive/functional status by 30 questions. The participant is asked to rank their own ability to perform different tasks independently on a 5-point scale from 1 (“cannot do”) to 5(“can do with ease”). Therefore, a higher score shows a perceived higher performance.
- PCRS-informant: elicits study partners’ perspective of the participants’ function using similar 30 questions worded appropriately for the informant. The study partner is asked to rank the participant’s ability on a similar 5-point scale.

PCRS has been extensively validated in numerous, large healthy and clinical patient datasets (39–41). Participants and their study partners responded to these questionnaires separately during a study visit with the supervision of trained study staff (study staff was available to confirm completion and to respond to potential questions during the test administration). We used the raw data from the participant and informant components of the questionnaire to calculate the anosognosia score (PCRS-self score minus PCRS-informant score). A participant was considered to have clinically meaningful anosognosia when their anosognosia score was above 20 or below −20 (20 points difference between patient’s and caregiver’s perspective of function), a previously published cutoff score for clinically meaningful anosognosia (42). We also categorized participants with clinically meaningful anosognosia to two groups of overestimators (patients who overestimated their own cognitive and functional abilities compared to their caregiver) and underestimators to explore the properties of this differential pattern in various dementia syndromes. We examined both the anosognosia score and clinically meaningful anosognosia status in distinct statistical analyses as appropriate.

#### Theory of mind

The Awareness of Social Inference Test: Social Inference-Enriched (TASIT-SIE) (43) was used to evaluate patients’ capacity for theory of mind. The TASIT-SIE is a face-to-face neuropsychological test that assesses an individual’s ability to detect insincere statements (i.e., lies and sarcastic remarks) in video vignettes featuring professional actors interacting with each other. Half of the items convey the insincerity via a visual cue and the other half use a verbal clue, facilitating the evaluation of how well a person can integrate different types of information to discern the protagonist’s true beliefs. After watching each vignette, participants answer four questions regarding the protagonist’s thoughts, intentions, and feelings. Test–retest reliability Convergence and discriminant validity have been previously evaluated in separate studies (9, 43) showing moderate to high test–retest reliability and strong validity for use in patients with early neurocognitive disorders (9) like those in our sample. We used the total score (max = 64) for our statistical analysis.

### Statistical analysis

All analyses were conducted in R (version 4.4.0: 2024-04-24) using the RStudio environment and the necessary packages including tidyverse, gtsummary, ggplot2, and MICE.

We described group differences in our sample on potentially confounding variables (age, sex assigned at birth, education, race/ethnicity) and included age and sex as confounders in our linear regression models. Our sample was very homogenous in education and race/ethnicity. The majority of our sample were highly educated and identified as White; therefore, we did not have meaningful variance to investigate the effect of race or education on the variables of interest and did not include these as potential confounders.

To describe the sample’s clinical and cognitive characteristics, we performed pairwise comparisons using the ANCOVA test and post-hoc correction for multiple comparisons controlling for confounds (Dunnett-Hsu) to evaluate the differences in our continuous variables (age, education, anosognosia score, theory of mind score, caregiver distress score) between dementia subtypes and control subjects represented in our sample. We used chi-squares for comparison of our categorical variables (sex, race/ethnicity).

For the regression analyses addressing our question of interest (the effects and interaction of anosognosia and ToM deficit on caregiver distress in dementia), we handled the missing values in our caregiver distress scores (*n* = 27), and theory of mind scores (*n* = 17) utilizing the Multivariate Imputation by Chained Equations (MICE) procedure. Variables with missing values were imputed under the assumption of missing at random (MAR), using predictive mean matching. A total of fifty imputed datasets were generated to ensure stable estimates.

We fitted three multivariable linear regression models separately:

1. Model 1: anosognosia score as a predictor for caregiver distress, controlling for age and sex.
2. Model 2: theory of mind score as a predictor of caregiver distress, controlling for age and sex.
3. Model 3: anosognosia and theory of mind scores as predictors of caregiver distress, controlling for age and sex.

We used Rubin’s rules to pool coefficients and obtain standard errors for inference. We then chose the best model fit that explained the highest amount of variance in caregiver distress as our final regression model (using adjusted R2 for comparison).

## Results

### Participant characteristics

Table 1 summarizes the characteristics of our sample (demographics and scores on our primary outcome variables within each clinical syndrome).

**Table 1:** Summary of descriptive analysis of sample characteristics.

Characteristics	Clinical syndrome						
	NC	AD	bvFTD	CBS	PSPS	PPA	svPPA
	*N* = 42	*N* = 57	*N* = 38	*N* = 14	*N* = 18	*N* = 12	*N* = 24
Age^a^	67.2 (8.58)	64.4 (10.34)	59.7 (10.61)**	63.8 (7.58)	66.4 (5.36)	66.9 (9.38)	62.9 (8.05)
Biological sex^b^							
Male	16 (38%)	35 (61%)	24 (63%)	6 (43%)	5 (28%)	5 (42%)	10 (42%)
Female	26 (62%)	22 (39%)	14 (37%)	8 (57%)	13 (72%)	7 (58%)	14 (58%)
Race/ethnicity^b^							
White	41 (98%)	56 (98%)	33 (87%)	13 (93%)	18 (100%)	10 (83%)	21 (87%)
Asian	0	1 (2%)	5 (13%)	1 (7%)	0	1 (8.5%)	2 (8.6%)
Other/Unknown	1 (2.4%)	0	0	0	0	1 (8.5%)	1 (4.4%)
Education (years)^a^	17.5 (1.99)	16.3 (2.57)	16.3 (3.15)	15.6 (2.22)	15.5 (3.07)	17.1 (4.53)	16.7 (2.61)
Anosognosia score (PCRS difference score)^a^	−5.3 (8.55)	10.9 (20.21)**	15.5 (26.57)***	11.9 (16.54)*	4.8 (18.86)	5.0 (20.33)	−1.1 (17.28)
Caregiver distress (NPI Distress Score)^a^	0.2 (1.12)	11.8 (10.50)***	15.4 (9.43)***	15.5 (13.34)***	16.0 (9.76)**	11.8 (6.97)***	12.9 (10.53)***
Theory of mind (social inference: TASIT-SIE)^a^	54.4 (5.21)	46.0 (5.79)***	47.2 (7.15)***	46.5 (6.33)***	47.4 (5.27)***	43.2 (6.13)***	38.9 (8.85)***

AD, Alzheimer’s disease syndrome; bvFTD, behavioral variant frontotemporal dementia syndrome; CBS, corticobasal syndrome; NC, cognitively normal control; PPA, non-fluent variant primary progressive aphasia syndrome and unspecified PPA; PSPS, progressive supranuclear palsy syndrome; svPPA, semantic variant primary progressive aphasia syndrome; NPI, Neuropsychiatric Inventory Questionnaire.

a Mean (sd).

b *n* (%).

Groups with statistically significant differences compared to controls are bolded for each variable (significance level: *p* < 0005:***, *p* < 005:**, *p* < 0.05*). ANOVA with Dunnett post-hoc test (for statistical significance adjustment for multiple comparisons) was used for continuous variables and chi-squared test for categorical variables.

On the NPI, all dementia groups showed significantly elevated levels of caregiver distress compared to informant reports on the healthy control group, and all dementia groups scored significantly worse on the TASIT-SIE social inference testing. The prevalence of clinically meaningful anosognosia [defined according to Sherer et al. (42) as >20 points difference between the estimate by the dementia patient versus that of their care partner] was significantly higher in all dementia syndromes compared to controls (prevalence compared to 2.4% for NCs *p* < 0.01: AD = 37%, bvFTD = 42%, CBS = 36%, PPA = 50%; *p* < 0.05: svPPA = 25%) except for PSPS (17%, *p* = 0.08). Separating anosognosia into two categories reflecting over- and under-estimation of function by the patients compared to their care partner, only the prevalence of overestimation was significantly higher in patients with dementia compared to controls (see Table 2).

**Table 2:** Clinically meaningful over−/under-estimation of function by patients across dementia syndromes.

Characteristics	Clinical syndrome						
	NC	AD	bvFTD	CBS	PSPS	PPA	svPPA
	*N* = 42	*N* = 57	*N* = 38	*N* = 14	*N* = 18	*N* = 12	*N* = 24
Estimation of function							
Accurate estimation	*N* = 41 (98%)	*N* = 36 (63%)	*N* = 22 (58%)	*N* = 9 (64%)	*N* = 15 (83%)	*N* = 6 (50%)	*N* = 18 (75%)
Overestimation	0	***N* = 18 (32%)**	***N* = 15 (39%)**	***N* = 5 (36%)**	***N* = 2 (11%)**	***N* = 4 (33%)**	***N* = 4 (17%)**
Underestimation	*N* = 1 (2%)	*N* = 3 (5%)	*N* = 1 (3%)	0	*N* = 1 (6%)	*N* = 2 (17%)	*N* = 2 (8%)

AD, Alzheimer’s disease syndrome; bvFTD, behavioral variant frontotemporal dementia syndrome; CBS, corticobasal syndrome; NC, cognitively normal control; PPA, non-fluent variant primary progressive aphasia syndrome and unspecified PPA; PSPS, progressive supranuclear palsy syndrome; svPPA, semantic variant primary progressive aphasia syndrome; NPI, Neuropsychiatric Inventory Questionnaire. Statistically significant between group differences are bolded (*p* < 0.001).

**Figure 1:**
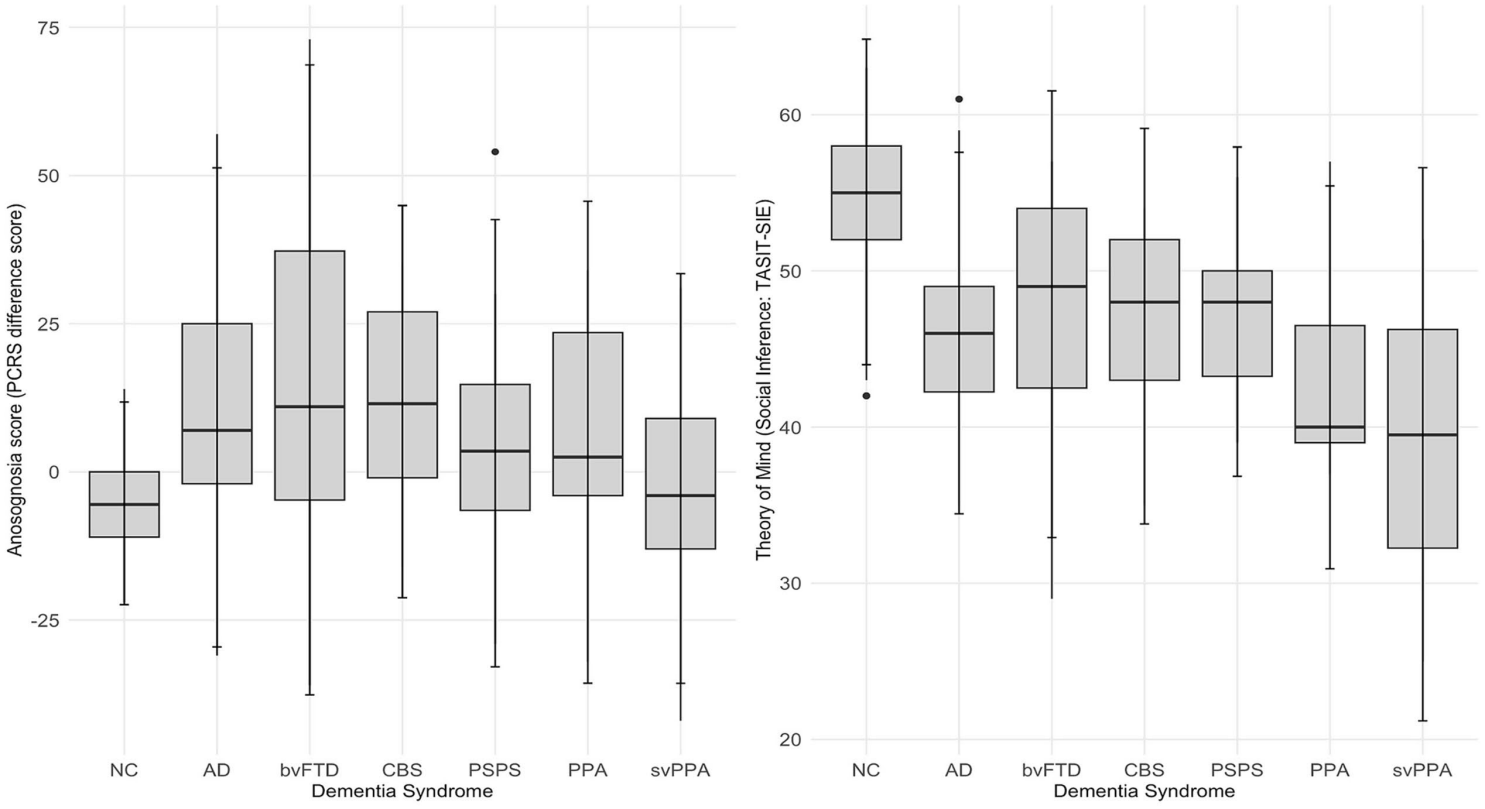
Anosognosia and theory of mind scores in various dementia syndromes. AD, Alzheimer’s disease syndrome; bvFTD, behavioral variant frontotemporal dementia syndrome; CBS, corticobasal syndrome; NC, cognitively normal control; PPA, non-fluent variant primary progressive aphasia syndrome and unspecified PPA; PSPS, progressive supranuclear palsy syndrome; svPPA, semantic variant primary progressive aphasia syndrome.

The relationship between anosognosia, theory of mind scores and dementia syndrome are illustrated in Figure 1.

### Regression analysis for caregiver distress prediction

**Figure 2:**
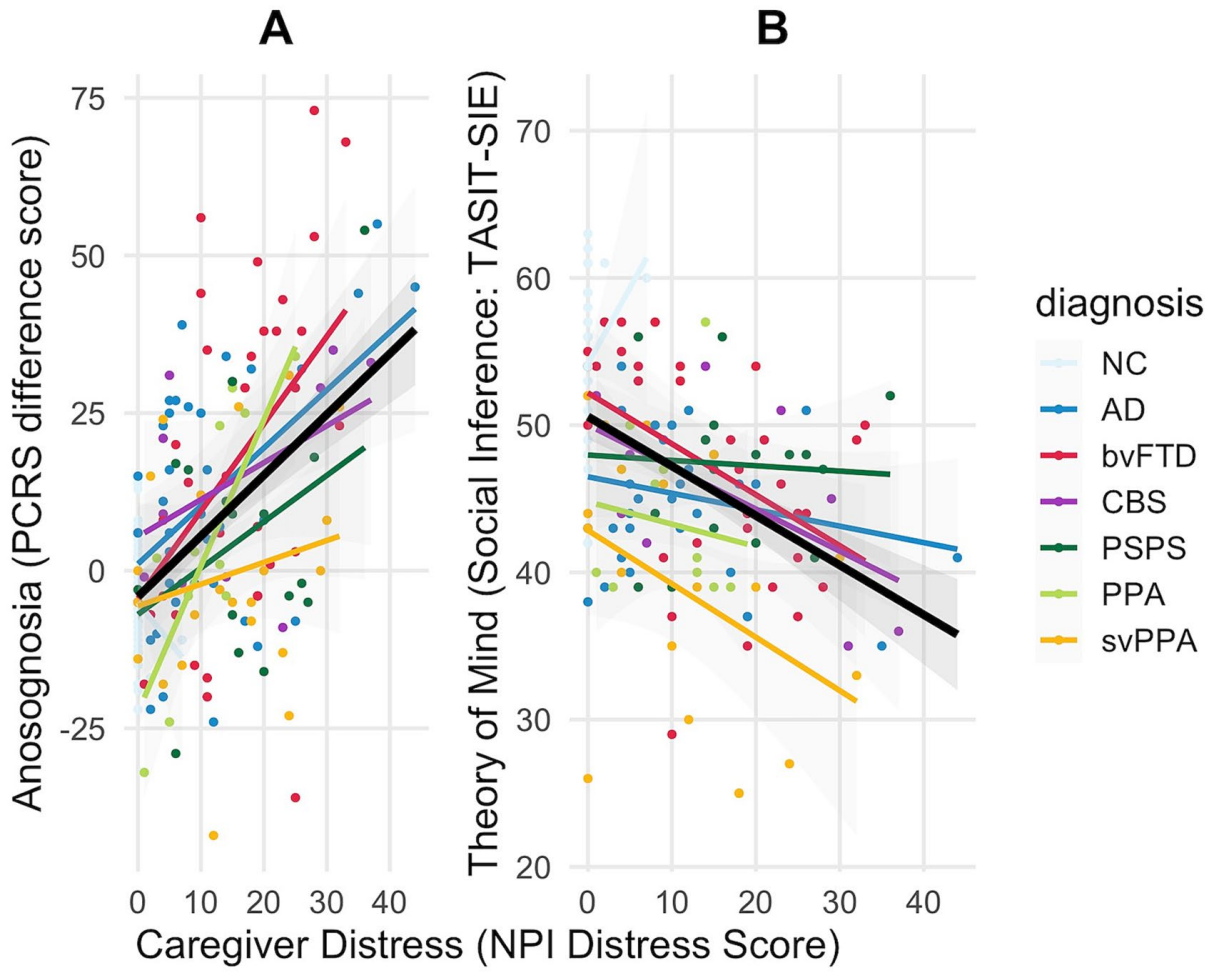
The relationship of anosognosia **(A)** and theory of mind **(B)** with caregiver distress in various dementia syndromes. AD, Alzheimer’s disease syndrome; bvFTD, behavioral variant frontotemporal dementia syndrome; CBS, corticobasal syndrome; NC, cognitively normal control; PPA, non-fluent variant primary progressive aphasia syndrome and unspecified PPA; PSPS, progressive supranuclear palsy syndrome; svPPA, semantic variant primary progressive aphasia syndrome. The black lines indicate the overall linear relationship between the predictor variable and caregiver distress regardless of dementia syndrome. Note that higher anosognosia score indicates worse performance in the Patient Competency Rating Scale (more severe anosognosia) and lower theory of mind score indicates worse performance in the Awareness of Social Inference Test-Social Inference Enriched test (more severe ToM deficit). This figure shows increased caregiver distress with worse patient performance in these measures.

First, separate models were created to determine whether anosognosia or theory of mind deficit significantly predicted caregiver distress. Both predictors significantly predicted caregiver distress, controlling for age and sex (see Table 3). There was a mild but statistically significant negative effect of sex in both models, such that caregivers of female patients with anosognosia or theory of mind deficit reported slightly lower distress compared to caregivers of men with similar anosognosia and theory of mind scores (*p* = 0.04 and *p* = 0.03 respectively) but in our final combined multivariable linear model, the sex effect was no longer statistically significant (*p* = 0.07). When both anosognosia and theory of mind deficit were combined as predictors in a multivariable linear regression model, both continued to independently predict caregiver distress after controlling for age and sex (Table 3; Figure 2). This final model showed the best fit for caregiver distress prediction, predicting 39% of variance in caregiver distress compared to models using anosognosia only (26%) and theory of mind only (23%). Each additional point higher anosognosia score (i.e., greater discrepancy between patients’ understanding of their own function with that observed by their caregiver) is associated with 0.21 higher caregiver distress score, and each additional point better social inference score (i.e., understanding of others’ perspective on the TASIT-SIE) is associated with 0.49 lower caregiver distress score.

**Table 3:** Multivariable linear regression model predicting caregiver distress.

Variables	Estimate	Standard error	Statistic	*p*-value
Intercept	38.17	5.91	6.46	<0001
Age (years)	−0.08	0.05	−1.22	0.2 (N.S)
Sex (female)	−2.27	1.25	−1.81	0.07 (N.S)
Anosognosia score (PCRS difference score)^a^	**0.21**	**0.03**	**6.55**	**<0001**
Theory of mind (social inference: TASIT-SIE)^b^	**−0.49**	**0.08**	**−6.98**	**<0001**

a Range in our sample: −42 to 73.

b Range in our sample: 25 to 63.

Statistically significant predictors are bolded. N.S, not significant.

The relationship of anosognosia and theory of mind scores with caregiver distress is illustrated in Figure 2.

## Discussion

In our study population of people with very mild stages of diverse dementia syndromes, we found that while both degree of patient anosognosia and theory of mind deficit were predictors of caregiver distress; modeling these patient factors together they accounted for 39% of the overall variance in caregiver distress, showing independent and additive contributions. We also showed that individuals with early-stage dementia, regardless of syndrome, were much more likely to overestimate their level of function relative to what caregivers observed and were not significantly likely to believe they had more deficits than actually observed. Moreover, patients with various dementia syndromes were significantly worse compared to controls in interpreting others’ intentions on theory of mind testing. This shows that while they can be coincident in some patients and thus have often been mistaken as two aspects of the same symptom, failure to correctly understand one’s own state (anosognosia) and failure to correctly understand another’s (ToM deficit) are distinct clinical issues and convey separate risks to the well-being of caregivers.

Our results overall are consistent with other studies examining caregiver distress when caring for PLwD with anosognosia and ToM deficit separately. Studies of caregiver burden in individuals with mild cognitive impairment (30), AD-type dementia syndrome (31) and bvFTD (24) have all found more caregiver burden in participants with anosognosia. The same has also been shown in a study focused on Huntington’s disease (44). However, our study further showed that when anosognosia is divided by whether the patient underestimates or overestimates their own function compared to the caregiver’s perception of reality, important new patterns emerge. Clinically meaningful overestimation and underestimation of function by the patient have previously been associated with different anatomical localization of cerebral atrophy (bilateral, right greater than left dorsal frontal gyri, orbitofrontal gyri, right anterior insula, putamen, thalamus, caudate, midbrain and pons for overestimation, and right rostral anterior cingulate for underestimation) (35). We found that clinically meaningful overestimation of function occurred in all of the dementia syndromes we evaluated, and the majority of patients did not significantly underestimate their abilities compared to controls. This overestimation, in turn, was the only direction of anosognosia that was predictive of caregiver distress. The fact that overestimation of function by PLwD causes conflict and distress for their caregivers is illustrated by the common example of driving, where caregivers are often placed in the position of needing to prevent the PLwD from driving against their will. This was thought to be “the biggest problem to face” by caregivers in a qualitative study investigating the effect of the need for driving cessation on caregivers of PLwD (45). In clinical practice, supervision of PLwD in order to prevent injury during daily activities such as cooking and self-care is another common area of conflict between PLwD who overestimate their abilities and their care partners, leading to distress for both. Knowing the significant effect of patient anosognosia/overestimation of function on caregivers is clinically important; therefore healthcare providers should probe for this important symptom when evaluating a person with dementia, regardless of syndrome. This can lead to early detection and appropriate counseling and support for the caregiver to more effectively cope with this phenomenon.

Theory of mind performance was also significantly worse in all syndromes of dementia in our study population compared to NCs and ToM deficit independently predicted caregiver distress, above and beyond anosognosia. While ToM deficit have repeatedly been shown in these patient groups, the relationship of ToM deficit to caregiver burden is less established. One study used the TASIT-SIE to evaluate ToM deficit in MCI, AD and bvFTD patients (10), and showed that greater ToM deficit predicted greater self-reported caregiver burden and distress. Misinterpretation of caregiver’s intentions by the PLwD is stressful for caregivers because it affects the interpersonal relationship between PLwD and their caregivers and in turn causes increased conflict and resistance to care. For example, the caregiver’s choice to monitor or restrict the PLwD’s cooking activities might be interpreted by the PLwD as a maliciously-intended interruption of autonomy and privacy. Furthermore, a PLwD with ToM deficit is also more likely to misinterpret the intentions and behaviors of others outside of the caregiving dyad, which can cause relational conflict and distress with friends, authorities, and strangers that the caregiver feels a burden to manage or resolve. Therefore, it is crucial for healthcare-providers to screen for this symptom and find dyads at risk of poor outcome related to this caregiver stressor.

The TASIT-SIE test has been used across clinical research studies to provide a valid measurement of ToM deficit in individuals with a variety of neurodegenerative syndromes, thus could be implemented if neuropsychological testing is an available option during clinical evaluation. However, in routine clinical care (46) with common time and expertise constraints it may not be feasible to perform formal neuropsychological testing during a brief evaluation for dementia (45). Other clinically brief evaluations of ToM deficit could be considered in such circumstances, including formulating clinical interview questions to be answered by the caregiver to describe if the patient tends to misunderstand the caregiver’s thoughts, feelings, or intentions. Given that our study confirms that ToM deficit has a direct, negative impact on caregiver distress, making an estimate of patients’ ToM deficit early in the dementia evaluation process can provide essential information to guide caregiver counseling and potentially inoculate them against toxic distress and burden.

Anosognosia and ToM deficit in our study had additive effect in predicting caregiver distress. This is a common clinical observation when interviewing caregivers of PLwD as a part of clinical management of dementia. A PLwD who overestimates their cognitive and behavioral function and misinterprets their caregiver’s intentions can be especially challenging to handle. For example, caregivers commonly report that their care-receiver who is not aware of their own difficulties with calculation and executive function (leading to poor money management), resist limitations of financial activities especially if they also misinterpret the concern reflected in their caregivers expressions about their risk of financial hardship as unnecessary and invasive meddling. Although no specific counseling plan for anosognosia or theory of mind deficit has yet been evaluated for effectiveness in a randomized clinical trial, more general psychosocial interventions focused on dementia caregivers have been shown to reduce caregiver distress (24, 25), thus incorporation of the interactive stressors of anosognosia and ToM deficit could be beneficial.

The effect of age as a patient-specific factor on caregiver distress is complex is confounded by the specific properties of the dementia syndromes more prevalent in young onset dementia (disease onset before age 65) compared to late onset. A study by Millenar and colleagues (47) comparing caregivers of young onset dementia and late onset dementia patients found that caregivers of young onset dementia patients were significantly younger and more commonly lived in the same household as the PLwD (90% vs. 54%). They reported poorer physical and psychological wellbeing in caregivers of young onset dementia cases. The frequency of the FTD syndromes is significantly higher in young onset compared to late onset dementia cases (12.7% compared to 1.9% in the Millenar sample) but the prevalence of AD or vascular dementia in their study population was not significantly different between the young onset and late onset dementia. It is worth noting that they only included caregivers of 30 patients with a mix of all FTD syndromes in their study, which is difficult to map to the dementia syndromes of interest in our study (47). Another study with a similar approach had similar findings (worse caregiver distress and symptoms in people caring for young onset dementia cases) (48). In our study, although participants with bvFTD were significantly younger than controls and other dementia syndromes, age of the PLwD was not significantly impactful in our linear regression models for prediction of caregiver distress. This is likely because we used age as a continuous variable rather than a binary variable.

## Limitations and conclusion

### Conclusion and implications for clinical practice

Our findings are consistent with other research showing that anosognosia in individuals with chronic neurological disorders including dementia can increase caregiver distress. Moreover, our study highlights the additive importance of patients’ ToM deficit to predict more caregiver distress above and beyond their anosognosia, emphasizing that evaluation of both patient anosognosia and ToM deficit in clinical contexts may provide meaningful information for identifying patient-caregiver dyads at higher risk of caregiver distress. This in turn can guide clinical practice and modify the adverse effects of these common dementia symptoms on the support network around the PLwD.

In line with our results, other studies have shown that other dementia-related symptoms affecting communication between patients and their care partners also exacerbate distress caregiver, for example difficulty with emotion recognition in patients with Huntington’s disease (49). Therefore, it is important to establish clinically useful screening tools and train healthcare providers to screen for those symptoms in every dementia clinical encounter to improve individualized dementia care. Furthermore, educating the broader community about the presence, etiology and potential consequences of such behavioral symptoms of dementia can impact the attitude of the community in encounters with PLwD and help with development of a dementia friendly environment that is understanding and supportive of PLwD and their care partners.

### Limitation and future direction

Our study is unique in evaluating the degree to which both patient anosognosia and ToM deficit impact caregiver distress in a variety of dementia subtypes but has limitations. Our sample was comparatively small, thus future multi-site studies with larger cohorts would be helpful to model the more complex interactions of anosognosia, ToM deficit, and caregiver burden with other social determinants of health, and within various dementia subtypes. For example, our sample was not designed to unequivocally determine whether caring for people with early onset dementia is associated with more distress compared to late onset dementia, thus additional work is needed with larger samples to investigate for differential impact of age of onset within syndrome. Our sample was restricted to people with mild stages of represented neurodegenerative diseases and therefore, the strength of the relationship observed between the patient variables of interest and caregiver distress might not necessarily be the same in more advanced stages of dementia. Our study could not examine the impact of additional psychiatric comorbidities on these relationships due to our limited sample size and available data. Similarly, we did not have data available from a formal measure of caregiver distress, such as the Lawton or Zarit burden measures, thus we could not more carefully examine distinct dimensions of caregiver burden to better understand the factors causing the greatest distress, thus these considerations are valuable topics for future investigations.

We did not include individuals with Parkinson’s disease dementia or Lewy body dementia in our study because of limited participant availability in our research cohorts. Considering the high prevalence of these syndromes in the general population, future investigation of our study questions in those dementia syndromes would provide valuable information for clinical practice. Finally, mechanistic studies incorporating brain structure and function might further clarify the complex relationships between anosognosia and ToM deficit. These potential studies can pave the way for designing anosognosia- and ToM deficit-specific interventions individualized to caregivers in order to improve outcomes.

## Data Availability

The raw data supporting the conclusions of this article will be made available by the authors, without undue reservation.

## References

[1] Alzheimer’s Association. Alzheimer’s Disease, Facts and Figures. (2025). Available online at: https://www.alz.org/getmedia/ef8f48f9-ad36-48ea-87f9-b74034635c1e/alzheimers-facts-and-figures.pdf (accessed Aug 4, 2025).

[2] Kandiah N Wang V Lin X Nyu MM Lim L Ng A . Cost related to dementia in the young and the impact of etiological subtype on cost. J Alzheimer's Dis. (2015) 49:277–85. doi: 10.3233/JAD-150471, 26444788

[3] Cheng ST. Dementia caregiver burden: a research update and critical analysis. Curr Psychiatry Rep. (2017) 19:64. doi: 10.1007/s11920-017-0818-2, 28795386 PMC5550537

[4] McKhann GM Knopman DS Chertkow H Hyman BT Jack CR Kawas CH . The diagnosis of dementia due to Alzheimer’s disease: recommendations from the National Institute on Aging-Alzheimer’s association workgroups on diagnostic guidelines for Alzheimer’s disease. Alzheimers Dement. (2011) 7:263–9. doi: 10.1016/j.jalz.2011.03.005, 21514250 PMC3312024

[5] Conde-Sala JL Reñé-Ramírez R Turró-Garriga O Gascón-Bayarri J Juncadella-Puig M Moreno-Cordón L . Clinical differences in patients with Alzheimer’s disease according to the presence or absence of anosognosia: implications for perceived quality of life. J Alzheimer's Dis. (2013) 33:1105–16. doi: 10.3233/JAD-2012-121360, 23128559

[6] Starkstein SE. Anosognosia in Alzheimer’s disease: diagnosis, frequency, mechanism and clinical correlates. Cortex. (2014) 61:64–73. doi: 10.1016/j.cortex.2014.07.019, 25481465

[7] Leocadi M Canu E Paldino A Agosta F Filippi M. Awareness impairment in Alzheimer’s disease and frontotemporal dementia: a systematic MRI review. J Neurol. (2023) 270:1880–907. doi: 10.1007/s00415-022-11518-9, 36512063

[8] Bastin C Giacomelli F Miévis F Lemaire C Guillaume B Salmon E. Anosognosia in mild cognitive impairment: lack of awareness of memory difficulties characterizes prodromal Alzheimer’s disease. Front Psych. (2021) 12:631518. doi: 10.3389/fpsyt.2021.631518, 33868048 PMC8044313

[9] Shany-Ur T Poorzand P Grossman SN Growdon ME Jang JY Ketelle RS . Comprehension of insincere communication in neurodegenerative disease: lies, sarcasm, and theory of mind. Cortex. (2012) 48:1329–41. doi: 10.1016/j.cortex.2011.08.003, 21978867 PMC3257415

[10] Formica C Bonanno L Todaro A Marra A Alagna A Corallo F . The role of mind theory in patients affected by neurodegenerative disorders and impact on caregiver burden. J Clin Neurosci. (2020) 78:291–5. doi: 10.1016/j.jocn.2020.05.028, 32402618

[11] Heitz C Noblet V Phillipps C Cretin B Vogt N Philippi N . Cognitive and affective theory of mind in dementia with Lewy bodies and Alzheimer’s disease. Alzheimer's Res Ther. (2016) 8:10. doi: 10.1186/s13195-016-0179-9, 26979460 PMC4793654

[12] Rosen HJ. Anosognosia in neurodegenerative disease. Neurocase. (2011) 17:231–41. doi: 10.1080/13554794.2010.522588, 21667396

[13] Gainotti G. Anosognosia in degenerative brain diseases: the role of the right hemisphere and of its dominance for emotions. Brain Cogn. (2018) 127:13–22. doi: 10.1016/j.bandc.2018.08.002, 30179807

[14] Valotassiou V Sifakis N Tzavara C Lykou E Tsinia N Kamtsadeli V . Anosognosia in dementia: evaluation of perfusion correlates using 99mTc-HMPAO SPECT and automated Brodmann areas analysis. Diagnostics. (2022) 12:1136. doi: 10.3390/diagnostics12051136, 35626292 PMC9140080

[15] Kipps CM Hodges JR. Theory of mind in frontotemporal dementia. Soc Neurosci. (2006) 1:235–44. doi: 10.1080/17470910600989847, 18633790

[16] Le Bouc R Lenfant P Delbeuck X Ravasi L Lebert F Semah F . My belief or yours? Differential theory of mind deficits in frontotemporal dementia and Alzheimer’s disease. Brain. (2012) 135:3026–38. doi: 10.1093/brain/aws237, 23065791

[17] Duval C Bejanin A Piolino P Laisney M De La Sayette V Belliard S . Theory of mind impairments in patients with semantic dementia. Brain. (2012) 135:228–41. doi: 10.1093/brain/awr309, 22232593 PMC3655376

[18] Rascovsky K Hodges JR Knopman D Mendez MF Kramer JH Neuhaus J . Sensitivity of revised diagnostic criteria for the behavioural variant of frontotemporal dementia. Brain. (2011) 134:2456–77. doi: 10.1093/brain/awr179, 21810890 PMC3170532

[19] Armstrong MJ Litvan I Lang AE Bak TH Bhatia KP Borroni B . Criteria for the diagnosis of corticobasal degeneration. Neurology. (2013) 80:496–503. doi: 10.1212/WNL.0b013e31827f0fd1 23359374 PMC3590050

[20] Höglinger GU Respondek G Stamelou M Kurz C Josephs KA Lang AE . Clinical diagnosis of progressive supranuclear palsy: the movement disorder society criteria. Mov Disord. (2017) 32:853–64. doi: 10.1002/mds.26987, 28467028 PMC5516529

[21] Gorno-Tempini ML Hillis AE Weintraub S Kertesz A Mendez M Cappa SF . Classification of primary progressive aphasia and its variants. Neurology. (2011) 76:1006–14. doi: 10.1212/WNL.0b013e31821103e6, 21325651 PMC3059138

[22] Stall NM Kim SJ Hardacre KA Shah PS Straus SE Bronskill SE . Association of informal caregiver distress with health outcomes of community-dwelling dementia care recipients: a systematic review. J Am Geriatr Soc. (2019) 67:609–17. doi: 10.1111/jgs.15690 30536383

[23] Hazzan AA Sniatecki JL Metz G Williams J. Alcohol use and abuse among family caregivers of people living with dementia in the United States: a scoping review. Int J Environ Res Public Health. (2024) 21:1525. doi: 10.3390/ijerph21111525, 39595792 PMC11594151

[24] Karnatz T Monsees J Wucherer D Michalowsky B Zwingmann I Halek M . Burden of caregivers of patients with frontotemporal lobar degeneration- a scoping review. Int Psychogeriatr. (2021) 33:891–911. doi: 10.1017/S1041610219000176, 30982478

[25] Kales HC Gitlin LN Stanislawski B Myra Kim H Marx K Turnwald M . Effect of the WeCareAdvisor™ on family caregiver outcomes in dementia: a pilot randomized controlled trial. BMC Geriatr. (2018) 18:113. doi: 10.1186/s12877-018-0801-8, 29747583 PMC5946471

[26] Kales HC Gitlin LN Lyketsos CG. Management of neuropsychiatric symptoms of dementia in clinical settings: recommendations from a multidisciplinary expert panel. J Am Geriatr Soc. (2014) 62:762–9. doi: 10.1111/jgs.12730, 24635665 PMC4146407

[27] Feast A Orrell M Russell I Charlesworth G Moniz-Cook E. The contribution of caregiver psychosocial factors to distress associated with behavioural and psychological symptoms in dementia. Int J Geriatr Psychiatry. (2017) 32:76–85. doi: 10.1002/gps.4447, 26891463

[28] Andrén S Elmståhl S. Relationships between income, subjective health and caregiver burden in caregivers of people with dementia in group living care: a cross-sectional community-based study. Int J Nurs Stud. (2007) 44:435–46. doi: 10.1016/j.ijnurstu.2006.08.016, 17078957

[29] Park MB Kim SM. The influence of cohabitation type on the psychological vulnerability of family caregivers of people with dementia: results from a community health survey of 324,078 people in Korea. Arch Gerontol Geriatr. (2022) 98:104558. doi: 10.1016/j.archger.2021.104558, 34717241

[30] Kelleher M Tolea MI Galvin JE. Anosognosia increases caregiver burden in mild cognitive impairment. Int J Geriatr Psychiatry. (2016) 31:799–808. doi: 10.1002/gps.4394, 26643996 PMC8483618

[31] Turrõ-Garriga O Garre-Olmo J Vilalta-Franch J Conde-Sala JL De Gracia BM Lõpez-Pousa S. Burden associated with the presence of anosognosia in Alzheimer’s disease. Int J Geriatr Psychiatry. (2013) 28:291–7. doi: 10.1002/gps.3824, 22555993

[32] Gallingani C Tondelli M Vannini P Zamboni G. The association between anosognosia and neuropsychiatric symptoms in neurodegenerative dementias: a narrative review. Front Neurol. (2025) 16:1649627. doi: 10.3389/fneur.2025.1649627, 41079349 PMC12511066

[33] Zegarra-Valdivia JA Rijpma MG Shany-Ur T Kramer JH Miller BL Rankin KP. Cognitive and emotional theory of mind in dementia. Impact on real life behaviors. Alzheimers Dement. (2023) 19:e067855. doi: 10.1002/alz.067855

[34] Spinelli EG Mandelli ML Miller ZA Santos-Santos MA Wilson SM Agosta F . Typical and atypical pathology in primary progressive aphasia variants. Ann Neurol. (2017) 81:430–43. doi: 10.1002/ana.24885, 28133816 PMC5421819

[35] Shany-Ur T Lin N Rosen HJ Sollberger M Miller BL Rankin KP. Self-awareness in neurodegenerative disease relies on neural structures mediating reward-driven attention. Brain. (2014) 137:2368–81. doi: 10.1093/brain/awu161, 24951639 PMC4107746

[36] Clare L Nelis SM Martyr A Whitaker CJ Marková IS Roth I . Longitudinal trajectories of awareness in early-stage dementia. Alzheimer Dis Assoc Disord. (2012) 26:140–7. doi: 10.1097/WAD.0b013e31822c55c4, 21909019

[37] Cummings JL. The neuropsychiatric inventory: assessing psychopathology in dementia patients. Neurology. (1997) 48:S10–6. doi: 10.1212/wnl.48.5_suppl_6.10s, 9153155

[38] Cummings JL Mega M Gray K Rosenberg-Thompson S Carusi DA Gornbein J. The neuropsychiatric inventory: comprehensive assessment of psychopathology in dementia. Neurology. (1994) 44:2308–14. doi: 10.1212/wnl.44.12.2308, 7991117

[39] Lin N Shany-Ur T Nguyen L Poorzand P Grossman SN Rosen HJ . The PCRS is a useful measure of anosognosia in neurodegenerative disease. J Am Geriatr Soc. (2012) 60:S60.

[40] Prigatano GP Altman IM. Impaired awareness of behavioral limitations after traumatic brain injury. Arch Phys Med Rehabil. (1990) 71:1058–64. 2256806

[41] Bivona U Costa A Ciurli P Donvito T Lombardi G Misici I . Modification of the patient competency rating scale to measure Anosodiaphoria after severe acquired brain injury: preliminary findings. Arch Clin Neuropsychol. (2022) 37:753–61. doi: 10.1093/arclin/acab096, 34933340

[42] Sherer M Hart T Nick TG. Measurement of impaired self-awareness after traumatic brain injury: a comparison of the patient competency rating scale and the awareness questionnaire. Brain Inj. (2003) 17:25–37. doi: 10.1080/0269905021000010113 12519645

[43] McDonald S Bornhofen C Shum D Long E Saunders C Neulinger K. Reliability and validity of the awareness of social inference test (TASIT): a clinical test of social perception. Disabil Rehabil. (2006) 28:1529–42. doi: 10.1080/09638280600646185, 17178616

[44] Wibawa P Zombor R Dragovic M Hayhow B Lee J Panegyres PK . Anosognosia is associated with greater caregiver burden and poorer executive function in Huntington disease. J Geriatr Psychiatry Neurol. (2020) 33:52–8. doi: 10.1177/0891988719856697 31213121

[45] Liddle J Tan A Liang P Bennett S Allen S Lie DC . The biggest problem we’ve ever had to face: how families manage driving cessation with people with dementia. Int Psychogeriatr. (2016) 28:109–22. doi: 10.1017/S1041610215001441, 26365085

[46] Zegarra-Valdivia JA Shany-Ur T Rijpma MG Callahan P Poorzand P Grossman S . Validation of the cognitive-emotional perspective taking test in patients with neurodegeneration. J Alzheimer's Dis. (2025) 104:436–51. doi: 10.1177/13872877251317683, 40026013 PMC12231792

[47] Millenaar JK De Vugt ME Bakker C Van Vliet D Pijnenburg YAL Koopmans RTCM . The impact of young onset dementia on informal caregivers compared with late onset dementia: results from the NeedYD study. Am J Geriatr Psychiatry. (2016) 24:467–74. doi: 10.1016/j.jagp.2015.07.005, 26560507

[48] Lim L Zhang A Lim L Choong TM Silva E Ng A . High caregiver burden in young onset dementia: what factors need attention? J Alzheimer's Dis. (2017) 61:537–43. doi: 10.3233/JAD-170409 29171995

[49] Zarotti N Storey A Lloyd S Guevara LM Caswell H Chen C . Emotion recognition in people with Huntington’s disease: a comprehensive systematic review. J Huntingtons Dis. (2025). doi: 10.1177/18796397251390252 PMC12847465 41166343

